# Can climatic factors explain the differences in COVID-19 incidence and severity across the Spanish regions?: An ecological study

**DOI:** 10.1186/s12940-020-00660-4

**Published:** 2020-10-13

**Authors:** Pedro Muñoz Cacho, José L. Hernández, Marcos López-Hoyos, Víctor M. Martínez-Taboada

**Affiliations:** 1 Gerencia de Atención Primaria, Servicio Cántabro de Salud, Santander, Spain; 2 Department of Internal Medicine, Hospital Marqués de Valdecilla-IDIVAL, Santander, Spain; 3 Facultad de Medicina, University of Cantabria, Santander, Spain; 4 Division of Immunology, Hospital Marqués de Valdecilla-IDIVAL, Santander, Spain; 5 Division of Rheumatology, Hospital Marqués de Valdecilla-IDIVAL, Santander, Spain

**Keywords:** Climatic factors, SARS-CoV-2 infection, COVID-19, Ultraviolet radiation, Temperature

## Abstract

**Background:**

Environmental factors play a central role in seasonal epidemics. SARS-CoV-2 infection in Spain has shown a heterogeneous geographical pattern This study aimed to assess the influence of several climatic factors on the infectivity of SARS-CoV-2 and the severity of COVID-19 among the Spanish Autonomous Communities (AA.CC.).

**Methods:**

Data on coronavirus infectivity and severity of COVID-19 disease, as well as the climatic variables were obtained from official sources (Ministry of Health and Spanish Meteorological Agency, respectively). To assess the possible influence of climate on the development of the disease, data on ultraviolet radiation (UVR) were collected during the months before the start of the pandemic. To analyze its influence on the infectivity of SARS-CoV-2, data on UVR, temperature, and humidity were obtained from the months of highest contagiousness to the peak of the pandemic.

**Results:**

From October 2019 to January 2020, mean UVR was significantly related not only to SARS-CoV-2 infection (cumulative incidence -previous 14 days- × 10^5^ habitants, *rho* = − 0.0,666; *p* = 0.009), but also with COVID-19 severity, assessed as hospital admissions (*rho* = − 0.626; *p* = 0.017) and ICU admissions (*rho* = − 0.565; *p* = 0.035). Besides, temperature (February: *rho* = − 0.832; *p* < 0.001 and March: *rho* = − 0.904; *p* < 0.001), was the main climatic factor responsible for the infectivity of the coronavirus and directly contributed to a different spread of SARS-CoV-2 across the Spanish regions.

**Conclusions:**

Climatic factors may partially explain the differences in COVID-19 incidence and severity across the different Spanish regions. The knowledge of these factors could help to develop preventive and public health actions against upcoming outbreaks of the disease.

## Background

Although we still do not know whether the SARS-CoV-2 pandemic will follow a seasonal pattern, several lines of evidence seem to support this possibility [1]. The appearance of the initial cases in China during the wintertime and the extraordinary spread of the infection during this period of the year are noted first [2]. Secondly, epidemics caused by other coronaviruses including SARS-CoV also occur during the winter months [3, 4]. In this sense, the fast spread of viral infection around the world, especially in temperate climates, resembles the similar seasonal pattern of other respiratory viral epidemics [5, 6]. Finally, based on epidemiologic models, a theoretical new wave of SARS-CoV-2 infection would be expected during the autumn/winter of 2020–2021 [7, 8].

If a seasonal pattern is expected, the analysis of environmental factors potentially involved in SARS-CoV-2 spreading, including the effect of climatic factors, might be an important issue to consider [4]. In fact, data on the influence of both temperature and humidity, reveals a clear association between these climatic parameters and virus survival and infectivity [9, 10]. Other factors, such as the amount of ultraviolet radiation (UVR), have not been explored in detail for SARS-CoV-2, but might be also critical to explain the epidemiological characteristics of this infection, as occur with other viral diseases [11, 12]. Although these environmental factors are not modifiable, some host defense mechanisms can interact or be directly related to them. In this regard, UVR has been used as a surrogate marker of vitamin D status in multiple studies [1, 13], and low UVR during the wintertime has been associated with the seasonal pattern of several viral infections [13, 14]. Vitamin D is a hormone related to multiple effects on the innate and acquired immune system and also involved in the proper production of antimicrobial peptides [13, 15]. Furthermore, there is some evidence to support the use of vitamin D supplements for the prevention of influenza or other respiratory viral infections [16].

Italy and Spain, two Southern European countries, have been hit by the COVID-19 pandemic in an extremely virulent way. Both are located in temperate zones of the Northern hemisphere, share a similar latitude, and have reached very high rates of SARS-CoV-2 infection and lethality [17]. Interestingly, SARS-CoV-2 infection in both countries has shown a heterogeneous geographical pattern. In fact, some differences between Autonomous Communities (AA.CC) in Spain are especially striking. Different factors might explain these regional differences, including human and environmental ones (5), and therefore, the assessment of climatic factors might provide a clue to deepen understanding about these differences and to design preventive measures.

Taking into account the above considerations, we aimed to assess the possible influence of UVR, temperature, and humidity on the incidence of SARS-CoV-2 infection and the severity of COVID-19.

## Material and methods

This is an ecological study (multiple-group design) [18], with data from different Spanish geographic areas correlating two variables: national epidemiological indicators of COVID-19 and three meteorological variables: UVR, temperature, and relative humidity.

### Data collection

Data on infectivity and severity of COVID-19 as well as climatic variables were obtained from national official sources (Ministry of Health; https://www.mscbs.gob.es) and Spanish Meteorological Agency (upon request: until March 31, 2020), respectively.

To assess the possible influence of the climatic factors on the development of the disease, data of UVR were collected during the months before the start of the pandemic in Spain (October 201 9-January 2020). UVR was measured in joules per square meter (J/m^2^), calculating the monthly averages of total radiation for the 4 months before the pandemic onset in Spain. Data on UVR was provided for 14 out of the 17 AA.CC. (official data provided were lacking for La Rioja, Navarra, and Asturias, which represents approximately 4.2% of the total Spanish population). We use the cumulative incidence (previous 14 days) × 10^5^ inhabitants, preceding March 30 as the infectivity indicator, the date when the peak of the incidence of new cases in Spain was reached.

To assess the possible influence on the infectivity of SARS-CoV-2 [9–11], data on UVR (J/m^2^), temperature (°C), and relative humidity (%) were also collected from the months with the highest infectivity (February and March 2020) to the peak of the pandemic in our country. For temperature and relative humidity, we used data from the same AA.CC of which we had UVR information. The meteorological variables of February and March have correlated with the cumulative incidence (previous 14 days) × 10^5^ inhabitants, on March 15 and April 15, respectively, to capture the whole impact of the previous month [19]. Data on cumulative incidence (previous 14 days) × 10^5^ inhabitants at the different time points of the study are shown in Supplementary Table 1.

### Statistical analysis

Climatic parameters (temperature, UVR, and relative humidity) were considered as independent variables and epidemiological variables related to COVID-19 (cumulative incidence during the previous 14 days, total cases, newly diagnosed cases, hospital admissions, intensive care unit (ICU) admissions, and mortality, all referring to the period from 1 to 30 March 2020) were analyzed as dependent variables. Linear regression models were built to assess the relationship between variables [20], and R^2^ was used to quantify the percentage of variation in the values of the dependent variable that can be explained by the variation in the independent variables. Spearman’s rho was used to analyze the correlation between variables. The Jonckheere-Terpstra test was used to determine the significance of a trend in the climatic data. A *p*-value < 0.05 was considered significant in all the calculations. All the statistical analysis of data was carried out with the IBM SPSS Statistics for Windows, version 25 (IBM Corp., Armonk, N.Y., USA).

## Results

**Fig. 1:**
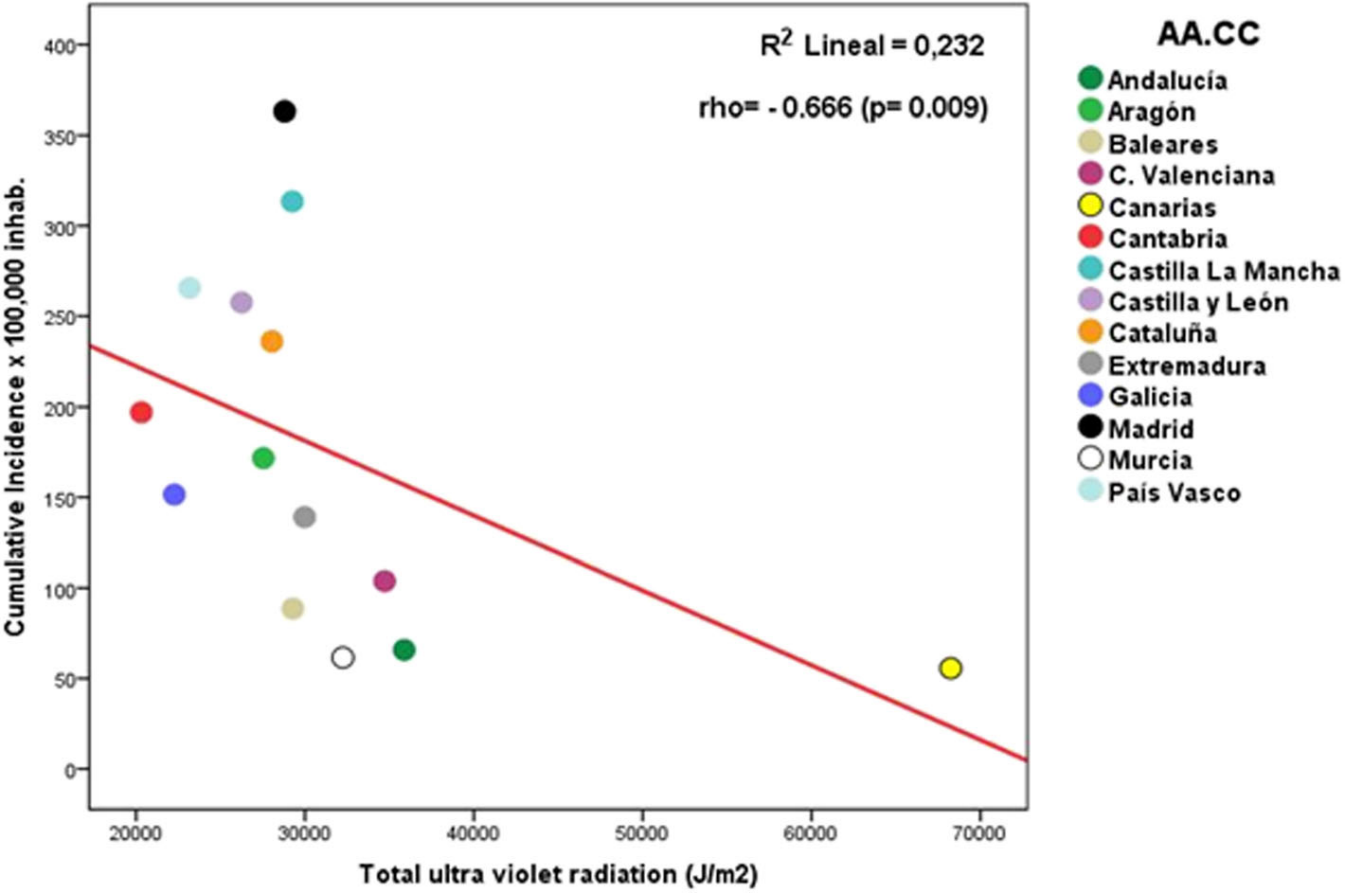
Relationship between cumulative UV radiation before the pandemic and cumulative incidence of SARS-CoV-2 infection across the Spanish AA.CC. Ultraviolet radiation was measured in joules per square meter (J/m^2^), calculating the monthly average total radiation for the 4 months (October to January) before the pandemic onset in Spain

As solar radiation, primarily acting through UVR, is the main factor involved in vitamin D synthesis [13], we first explore the accumulated dose of UVR during the months before the start of the pandemic in Spain. The average UVR from October 2019 to January 2020 was significantly related to SARS-CoV-2 infection (cumulative incidence during the previous 14 days, *rho* = − 0.666; *p* = 0.009) (Fig. 1 and Table 1). Regions with the highest accumulated UVR radiation during the previous months displayed the lowest cumulative incidence of SARS-CoV-2 infection. The association between UVR and SARS-CoV-2 infection remained significant after excluding the Canary Islands (a Spanish region located at a quite different latitude than Southern Spain) (Table 1 and Supplementary Figure 1). Furthermore, accumulated UVR was also related to COVID-19 severity assessed as hospital admissions (*rho* = − 0.626; *p* = 0.017) and ICU admissions (*rho* = − 0.565; *p* = 0.035) (Table 1).

**Table 1:** Relationship between total UVB radiation from October 2019 to January 2020 and epidemiological and severity parameters of COVID-19 disease × 100,000 inhabitants in the Spanish Autonomous Communities (AA.CC.) with official data

COVID-19 parameter	14 AA.CC.			13 AA.CC.		
	*Rho*	*p*	*R2*	*Rho*	*p*	*R2*
**Total cases**	−0.666	**0.009**	0.203	−0.582	**0.037**	0.157
**Cumulative incidence**^**a**^	−0.666	**0.009**	0.203	−0.582	**0.037**	0.195
**New cases**^**b**^	−0.508	0.064	0.109	−0.451	0.122	0.069
**Hospital admission**	−0.626	**0.017**	0.148	−0.549	0.052	0.112
**ICU admission**	−0.565	**0.035**	0.109	−0.478	0.098	0.069
**Deaths**	−0.446	0.110	0.060	−0.324	0.280	0.005

^a^Cumulative incidence (previous 14 days) per 100,000 inhabitants

^b^Accumulated data from the pandemic onset until March 30, 2020

14 AA.CC: Autonomous Communities in Spain without La Rioja, Navarra, and Asturias (see Material and Methods); 13 AA.CC: all Autonomous Communities in Spain with data available excluding the Canary Islands

Previous studies have suggested that both temperature and humidity are relevant for viral survival and infectivity [10, 20] and, therefore, we have also explored whether differences in these factors among the Spanish AA.CC. might explain the different incidence of COVID-19 in Spain. Furthermore, direct UVR might also contribute to viral load and inactivation, although data for SARS-CoV on this matter are scarce [21].

The impact of climatic conditions on SARS-CoV-2 infectivity was studied independently during February and March 2020 for several reasons. Firstly, and from a climatological point of view, both months have been characterized by atypical weather conditions in Spain. Thus, February has been a drier and warmer month than usual and, on the other hand, March had a particularly high rainfall. Besides, February can more accurately reflect the influence of meteorological variables on infectivity after the arrival of SARS-CoV-2 infection in Spain from abroad. However, in March, in addition to having increased person-to-person transmission, the impact of outdoor conditions could be less relevant due to the strict confinement measures imposed on March 13, 2020.

**Fig. 2:**
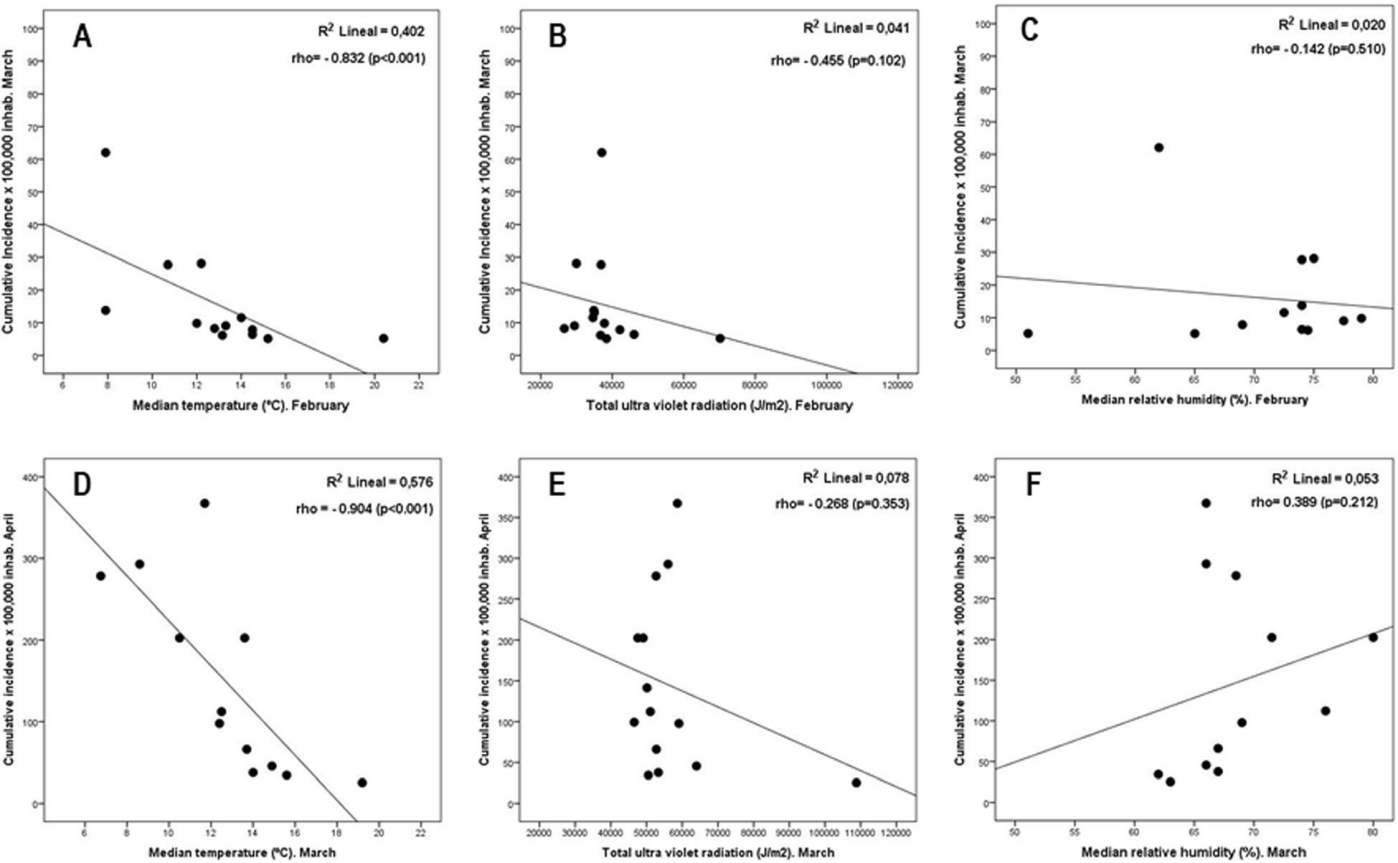
Relationship between climatic factors and infectivity of SARS-CoV-2 across the Spanish AA.CC. Data on UVR (J/m^2^), temperature (°C), and relative humidity (%) were collected from the months with the highest infectivity. These meteorological variables were correlated with the cumulative incidence (previous 14 days) × 10^5^ inhabitants, on March 15 for February climate variables and on April 15 for March climate variables. *p < 0.001

As shown in Fig. 2, the main climatic factor responsible for the differences in SARS-CoV-2 infection (in terms of cumulative incidence during the previous 14 days × 10^5^ habitants) was the temperature, both during February (*rho* = − 0.832; *p* < 0.001) and March (*rho* = − 0.904; *p* < 0.001). Spanish regions with the highest temperature had the lower incidence of infection (Fig. 2a and d). However, statistical significance of the association with SARS-CoV-2 infectivity was not reached for direct UVR (Fig. 2b and e) and relative humidity (Fig. 2c and f).

**Fig. 3:**
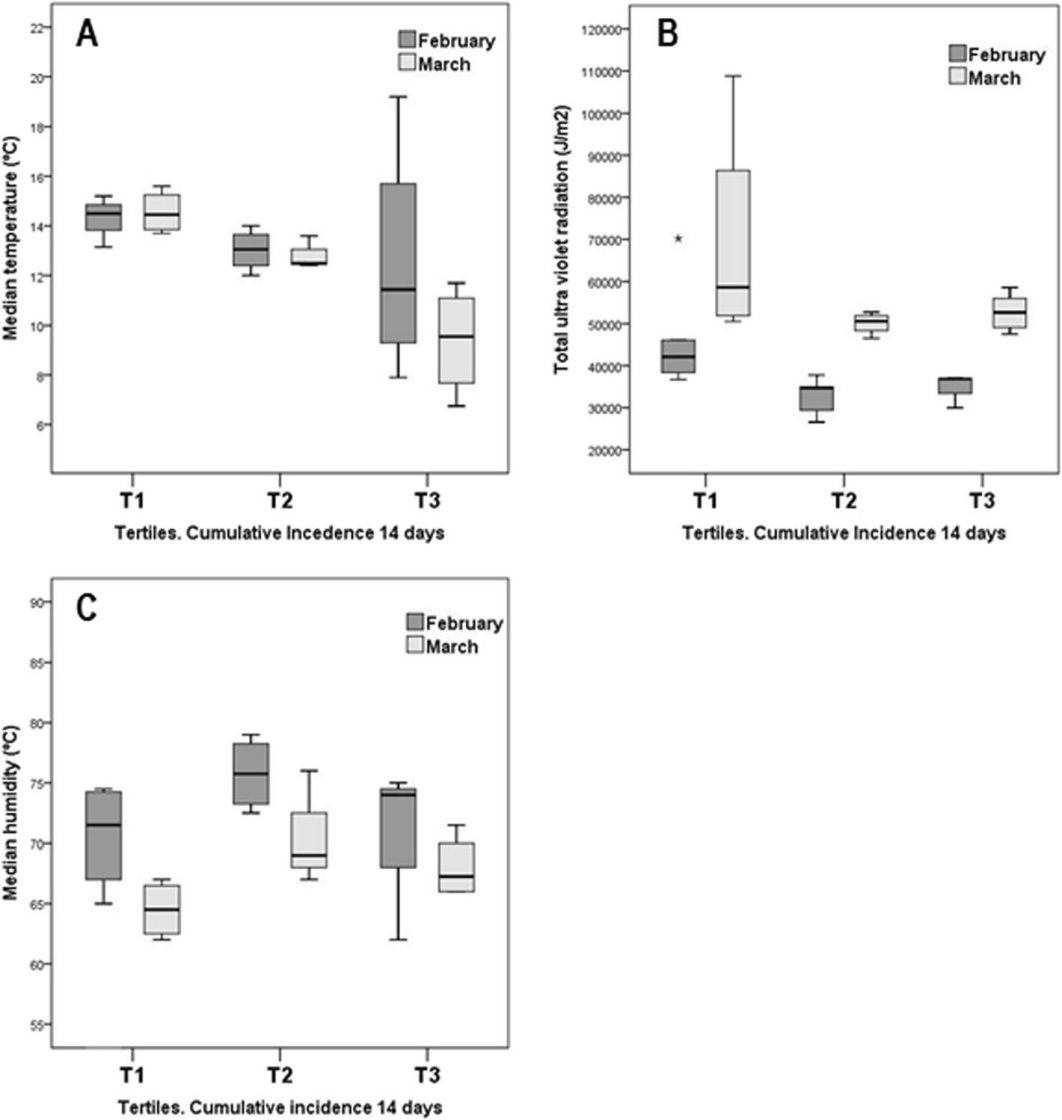
Relationship between climatic factors and infectivity across the Spanish AA.CC grouped according to tertiles of cumulative incidence of SARS-CoV-2. Data on UVR (J/m^2^), temperature (°C), and relative humidity (%) were collected from the months with the highest infectivity (February and March 2020). T1, T2, and T3 tertiles refer to AA.CC with low, medium, or high cumulative incidence (previous 14 days) × 10^5^ inhabitants, of SARS-CoV-2 infection. Statistical differences* (p) in tertiles of the cumulative incidence in the two periods analyzed, for each of the meteorological variables

Additional evidence for a hierarchy in climatic factors, depending on the time of infection, is shown in Fig. 3. As can be seen, the temperature (Fig. 3a), both at the beginning of the infection and during March 2020, shows a significant association with SARS-CoV-2 infection. On the other hand, during the same month, and despite increased UV radiation, no significant effect on infectivity was clearly shown (Fig. 3b). Again, we found no significant differences regarding relative humidity according to the tertiles of SARS-Cov-2 infection (Fig. 3c).

## Discussion

Our study shows that differences in SARS-CoV-2 infection rates and COVID-19 severity across Spanish regions are related to UVR exposure in the preceding months. Furthermore, temperature seems to also play a crucial role in SARS-CoV-2 infectivity and could explain, at least in part, the differences across the different AA.CC.

The two major contributing factors for the seasonal pattern of the respiratory viruses are the changes in the environmental parameters and human behavior [5]. Here, we show data that provide a clue to understanding one of the main environmental parameters, such as climatic conditions. Thus, regardless of other relevant factors, we found that low UVR in the months preceding the pandemic could explain, at least in part, the differences in the infectivity and severity of SARS-CoV-2 infection across the different Spanish regions. The possible influence of UVR in the severity of SARS-CoV-2 infection might be related to the selection of patients tested for SARS-CoV-2 in Spain, although the role of sun exposure should not be discarded. In fact, UVR is the main source of vitamin D (13), and it is tempting to speculate that vitamin D deficiency could predispose individuals to SARS-CoV-2 infection. Without a high dietary (or supplemental) intake of vitamin D, sun exposure is essential to avoid vitamin D insufficiency. Furthermore, previous studies suggest that low serum 25-hydroxyvitamin D (25OHD) levels are associated with an increased risk of acute respiratory infections in a dose-dependent manner and irrespective of age [22].

The accumulated UVR before starting the pandemic was not only associated with the rate of SARS-CoV-2 infection but also significantly related to the severity of the disease, in terms of hospitalization and ICU admission. In Spain, the majority of serious cases have affected elderly patients, those with associated comorbidities and, with special virulence, nursing home residents (Ministry of Health; https://www.mscbs.gob.es). In a great number of these individuals, serum 25OHD levels are usually low [23–26], and therefore might contribute to the exceedingly high COVID-19 severity and lethality they present. Besides, vitamin D deficiency has been found to also contribute to acute respiratory distress syndrome [27], the most frequent severe complication related to COVID-19 mortality [28]. Preliminary data from our group suggests that patients with COVID-19 have especially low levels of circulating 25OHD at hospital admission (data not shown). Although we do not have information on serum 25OHD levels in patients with a mild disease not requiring hospitalization, advice on personal sun exposure or treatment with vitamin D supplements (if needed) might be an easy, cheap and effective option to decrease SARS-CoV-2 impact.

On the other hand, several studies have pointed out the role of climatic factors on the SARS-CoV-2 infectivity. In this regard, the impact of temperature and humidity on respiratory virus stability and transmission rates seems to be crucial (5). Furthermore, as previously reported, heating and UVR can efficiently eliminate the viral infectivity of other SARS coronaviruses [21]. Therefore, investigating the effect of these climatic aspects on SARS-CoV-2 infection might also provide a clue to explain the differences in the spread of new cases of COVID-19 across the Spanish regions. We have found, in agreement with previous reports [4], that cold temperature usually provides a conducive environmental condition for virus survival. Here, we demonstrate that temperature is the most relevant factor in explaining the extrinsic variation in the survival of the virus.

Although there are no data on the impact of UVR on the infectivity of SARS-CoV-2, the trend towards a possible role of UVR during February (*rho* = − 0.455; *p* = 0.102), could suggest that in months with higher solar radiation, it could have a certain effect on it. Despite a higher UVR during March, the data may be masked by the confinement measures imposed by the Spanish Government. It is interesting to highlight that changes in host behavior, especially more time spent indoors, might also be followed by a less relevant role of some outdoor factors [29].

Although low humidity seems to be a more critical environmental factor influencing the outbreak of human coronavirus disease in China [4, 10], our results in Spain did not show the same pattern. These divergences with the data reported from China may have several explanations. Thus, the narrow range of relative humidity in Spain during this winter might not allow detecting this effect of humidity on the risk of infection. Besides, we do not have data on absolute humidity, which, as some authors have suggested, may be a better indicator of the impact of this climatic variable [19]. As previously stated, this divergence might be the result of differences in temperature or other climatic factors, and also of changes in host behavior induced by the pandemic itself and by the imposed confinement measures. Wintertime in temperate climates is usually linked to more time spent indoors where contacts are closer. Furthermore, the number of people-to-people contacts significantly increases on indoor settings during the progression of the pandemic, especially in hospitals and nursing homes, the two places that include the population hardest hit by the SARS-CoV-2 pandemic in Spain (elderly people and health care workers), while local weather conditions might have minor effects on the contacts. This seasonal and confinement situation also translates into low indoor relative humidity, which might also accelerate the spread of the virus [5]. Finally, contradictory results regarding the influence of climatic factors in other countries have also been obtained with other coronaviruses, such as MERS-SARS [30, 31]. Thus, it could be possible that viral infectivity might change depending on the particular climate conditions of each location.

Our study has several limitations. Firstly, data on patients infected with SARS-CoV-2 are obtained from the Spanish Government official sources. This data might underestimate the real data on infection as only patients with more symptomatic and severe manifestations have been tested up to now. However, we consider that this aspect does not influence the findings of this study since the same national policy has been applied in all Spanish regions since the start of the pandemic. Secondly, the number of hospitalizations and ICU admissions are more reliable markers of disease severity, although the inverse association of severity and UVR might also reflect the selection of only moderate to severe COVID-19 symptomatic cases. Nevertheless, the number of deaths might also be underestimated, since many deaths that occur outside of hospitals have probably not been accounted as related to SARS-CoV-2 infection. Thirdly, epidemiological data might have been affected by the time of interventions since March 13, 2020, when the alarm status was imposed by the Spanish Government. However, it is possible that the confinement of the population did not have a relevant effect on our results of accumulated UVR impact since the analysis was performed with data from April 1, 2020 and, given the incubation period of the disease, the impact on the results has likely been minimal. Thirdly, official climatic data on three regions of Spain (La Rioja, Navarra, and Asturias) were not provided, but we thought that the findings we have presented provide also relevant information as representing more than 95% of the Spanish population. Finally, a possible limitation of the study is that the so-called ecological fallacy may have been committed, that is to failure in reasoning that arises when an inference is made about an individual based on aggregate data for a group. Therefore, the design used serves to propose hypotheses that must be corroborated with other epidemiological designs.

## Conclusions

In summary, the results of our study support the influence of climatic factors on the incidence and severity of SARS-COV-2 infection and might explain, at least in part, the differences observed across the different Spanish regions. Thus, meteorological information should be integrated into a future forecast of a potential new outbreak of SARS-CoV-2. If the influence of UVR on circulating serum 25OHD levels in patients with COVID-19 is confirmed, the potential preventive treatment with vitamin D supplementation in high-risk groups for SARS-CoV-2 infection, especially in those individuals with vitamin D deficiency, merits further investigation in well-designed randomized controlled trials.

## Supplementary information

**Additional file 1: Supplementary Table 1.** Cumulative incidence -previous 14 days- × 10^5^ inhabitants in the different study periods across the Spanish regions.

**Additional file 2: Supplementary Figure 1.** Relationship between cumulative UV radiation before the pandemic and cumulative incidence of SARS-CoV-2 infection across the Spanish AA.CC. except Canary Islands.

## Data Availability

National official sources (Ministry of Health; https://www.mscbs.gob.es/) and Spanish Meteorological Agency (upon request: http://www.aemet.es/),

## Abbreviations

AA.CC.: Spanish Autonomous Communities
UVR: Ultraviolet ration
ICU: Intensive Care Unit
25OHD: 25-hydroxyvitamin D
