# Supplementary material for: Can climatic factors explain the differences in COVID-19 incidence and severity across the Spanish regions?: An ecological study
Source: Environ Health. 2020 Oct 13;19:106. doi: 10.1186/s12940-020-00660-4 (PMC7552591; doi:10.1186/s12940-020-00660-4)
Supplement: Supplementary file 1 — Additional file 1: Supplementary Table 1. Cumulative incidence -previous 14 days- × 105 inhabitants in the different study periods across the Spanish regions. [file 12940_2020_660_MOESM1_ESM.docx]

**Supplementary Table 1.** Cumulative incidence -previous 14 days- x10^5^ inhabitants in the different study periods across the Spanish regions.

| **AA.CC.** | **March 1** | **March 15** | **March 31** | **April 15** |
| --- | --- | --- | --- | --- |
|  |  |  |  |  |
| **Andalucía** | 0.14 | 6.44 | 65.76 | 45.58 |
| **Aragón** | 0.00 | 13.19 | 171.68 | 141.44 |
| **Baleares** | 0.17 | 6.18 | 88.65 | 37.67 |
| **Canarias** | 0.33 | 5.20 | 55.68 | 25.26 |
| **Cantabria** | 0.17 | 8.26 | 197.05 | 99.30 |
| **Castilla La Mancha** | 0.05 | 27.74 | 313.45 | 367.41 |
| **Castilla León** | 0.13 | 13.79 | 257.51 | 292.76 |
| **Cataluña** | 0.12 | 11.57 | 236.15 | 202.60 |
| **C. Valenciana** | 0.30 | 7.87 | 103.84 | 66.09 |
| **Extremadura** | 0.37 | 9.83 | 139.08 | 97.78 |
| **Galicia** | 0.00 | 9.08 | 151.55 | 112.28 |
| **Madrid** | 0.21 | 62.07 | 363.22 | 278.22 |
| **Murcia** | 0.00 | 5.15 | 61.52 | 34.41 |
| **País Vasco** | 0.14 | 28.13 | 265.65 | 202.60 |

AA.CC: Autonomous Communities
