# Supplementary figures and images for: Can climatic factors explain the differences in COVID-19 incidence and severity across the Spanish regions?: An ecological study
Source: Environ Health. 2020 Oct 13;19:106. doi: 10.1186/s12940-020-00660-4 (PMC7552591; doi:10.1186/s12940-020-00660-4)

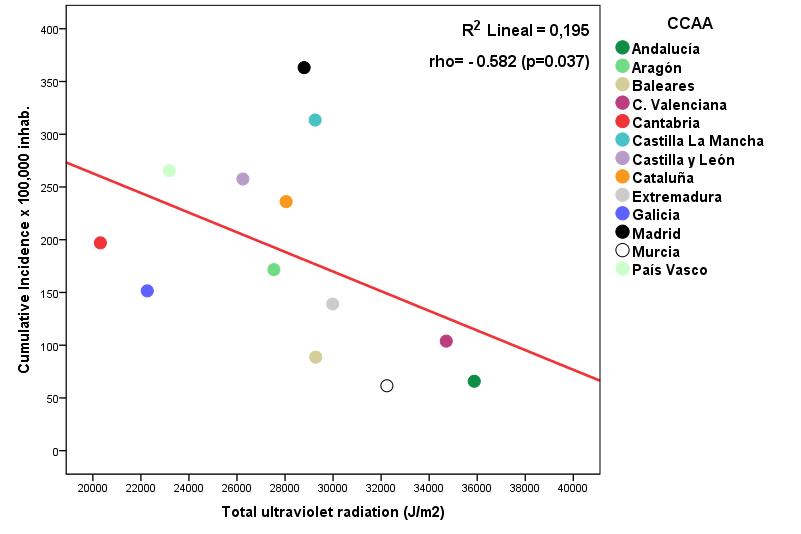

Supplement: Supplementary file 2 — Additional file 2: Supplementary Figure 1. Relationship between cumulative UV radiation before the pandemic and cumulative incidence of SARS-CoV-2 infection across the Spanish AA.CC. except Canary Islands. [file 12940_2020_660_MOESM2_ESM.docx]
